# Microbial Consortium Associated with Crustacean Shells Composting

**DOI:** 10.3390/microorganisms10051033

**Published:** 2022-05-16

**Authors:** Svetlana N. Yurgel, Muhammad Nadeem, Mumtaz Cheema

**Affiliations:** 1 USDA-ARS, Grain Legume Genetics and Physiology Research Unit, Prosser, WA 99350, USA; 2 School of Science and the Environment, Grenfell Campus, Memorial University of Newfoundland and Labrador, Corner Brook, NL A2H 5G4, Canada; mnadeem@grenfell.mun.ca (M.N.); mcheema@grenfell.mun.ca (M.C.)

**Keywords:** composting, sustainable agriculture, crustacean shells, microbial communities

## Abstract

Soil microbes play an essential role in the biodegradation of crustacean shells, which is the process of sustainable bioconversion to chitin derivatives ultimately resulting in the promotion of plant growth properties. While a number of microorganisms with chitinolytic properties have been characterized, little is known about the microbial taxa that participate in this process either by active chitin degradation or by facilitation of this activity through nutritional cooperation and composting with the chitinolytic microorganisms. In this study, we evaluated the transformation of the soil microbiome triggered by close approximation to the green crab shell surface. Our data indicate that the microbial community associated with green crab shell matter undergoes significant specialized changes, which was reflected in a decreased fungal and bacterial Shannon diversity and evenness and in a dramatic alteration in the community composition. The relative abundance of several bacterial and fungal genera including bacteria *Flavobacterium*, *Clostridium*, *Pseudomonas*, and *Sanguibacter* and fungi *Mortierella*, *Mycochlamys*, and *Talaromyces* were increased with approximation to the shell surface. Association with the shell triggered significant changes in microbial cooperation that incorporate microorganisms that were previously reported to be involved in chitin degradation as well as ones with no reported chitinolytic activity. Our study indicates that the biodegradation of crab shells in soil incorporates a consortium of microorganisms that might provide a more efficient way for bioconversion.

## 1. Introduction

Globally, around 600,000 tons of chitin waste are generated by the seafood industry annually [1], which brings substantial challenges for waste disposal; the disposal of one ton of shellfish waste through disposal outlets can cost up to USD 150 [2]. The routes on the crustacea waste disposal require a third party or company to take the material away from the possessing site for disposal by methods including aerobic and aerobic digestion, composting, and land spreading. Nevertheless, the reuse of waste from the industry is not a common practice, and a significant proportion of crustacean waste is deposited into the environment, generating potential environmental hazards due to fish tissue deterioration. Importantly, crustacean shells contain chitin, proteins, calcium carbonate, and carotenoids, which are valuable resources for pharmaceutical, agricultural, construction, and paper industries [1,2,3,4,5]. For example, treatments with chitin and shell derivatives have been shown to increase soil suppressiveness against plant fungal and nematode infections [6,7,8,9,10] and therefore attract significant attention as potential environmentally friendly approaches to control plant pathogens. Additionally, shell chitin derivatives are applied in agriculture to improve plant growth by influencing plant growth-promoting microorganisms, stimulating plant metabolism [11,12], and the induction of defense-related genes [7,13].

Chitin (polymer of (1→4)-β-linked N-acetyl-D-glucosamine) constitutes 15% to 40% of crustacean exoskeletons, representing around 75% of the shell organic fraction [14]. Industrial chitin extraction through chemical means has a number of limitations, including high energy requirements and the involvement of large amounts of sodium hydroxide and acidic treatments [15]. The biological extraction of chitin is a promising process, which can provide an alternative solution for decreasing production and environmental costs [1]. This process involves several steps, such as deproteinization, fermentation, and chitin-to-chitosan conversion, and could be facilitated by a number of microorganisms [7,16,17,18].

Culture-dependent and -independent analysis of chitinolytic microorganisms indicated that bacteria affiliated with *Actinomyces*, *Proteobacteria*, *Flavobacteria*, and *Firmicutes* and fungi *Aspergillus* and *Mortierella* are often identified as active chitin degraders [7,19,20,21]. More specifically, labeling studies pointed to a few key chitinolytic taxa, such as *Pseudomonas*, *Massilia*, and several families of *Bacteroidetes* as the most active in chitin degradations [22]. It was also suggested that chitinolytic bacteria sometimes process more chitin polymers than they are able to use themselves [23,24], providing the excess of the product to “satellite microbes”, opening the possibility for interspecific cross-feeding [19,22], a complex microbial cooperation within the chitin-degrading community. This in turn can improve the efficiency of chitin hydrolysis. The understanding of this already complex process is further complicated by the fact that chitin-degrading communities can undergo a temporal fluctuation [20] over a relatively short period of time: five to 90 days. Moreover, most of the studies aiming to identify chitinolytic microorganisms were focused on the soil bulk microbiome, while the analysis of those more tightly associated with shell microbes might provide additional information about the microbial complex involved in shell biodegradation. This approach could provide additional tools to facilitate environmentally friendly shell waste degradation.

The green crabs (*Carcinus aestuarii*) were first found in the 1950s in the eastern province of New Brunswick and extended to the southern part of Halifax in 1952. Since then, the population continued growing, and extended all over Newfoundland and Prince Edward Island [25]. The researchers worked on chitin extracted from green crab biomass to generate biodegradable polymers [26], and specialized chitin-based growing media had shown a reduction in soil pathogen levels leading to improved vegetable production [26]. The green crabs also had shells rich in calcium that can be used to neutralize acidic soils in Atlantic Canada. Similarly, the extracted chitin from crabs had a wide range of applications in Biomedicine, Pharmaceuticals, Food, Agriculture, and Personal Care Products [27]. The chitin-based compost could be used directly as a growing horticultural media to enhance soil pathogen control and significantly improve crop growth or as an extraction source for chitin/chitosan to be used for high-value applications in pharmaceuticals and the agricultural sector [27]. Previously, the crustacean composts were used in the nitrogen fixation, making the nitrogen available directly to the plants when added to the roots. The pathogens and microorganisms present in the crustaceans led to the degradation of the pathogens’ cell walls using the chitinases by composting [28].

The goal of this research was to assess the microbial communities, to evaluate the bacterial and fungal community tightly associated with green crab shell composted in Newfoundland, Canada, soils for a 1-year period, and to identify the microbial complex of potential chitin degraders.

## 2. Materials and Methods

### 2.1. Sampling Site Description and Sample Collection

The samples were collected on 14 October 2019, from Black Duck Siding, Western Newfoundland, Canada (48°34′00.1″ N 58°22′36.4″ W). The shells were originated from green crab processing facilities in Stephenville NL and were buried in soils for 1 year as part of the routine shell composting. A quantity of 15 crab shells (shell samples) were unearthed by a spatula from the soil at different locations on the same study site and packed in the labeled plastic bags, whereas the adherent soil (soil samples) to the crab shells was also collected in the separate plastic bags. The spatula was cleaned between different sample collections to avoid any cross-contamination. A quantity of 15 control soil (control) samples from the adjacent location of the collection site were also collected at the same time. All the collected samples were then placed on ice and transported to the laboratory for processing. After transportation to the lab, 5 g of the soil samples was sieved (2 mm) and immediately stored at −86 °C until processing for DNA isolation. The shells were removed from the bags, vigorously shaken, frozen in liquid nitrogen, ground into a fine powder using a sterile pestle, and stored at −86 °C.

### 2.2. DNA Extraction and Sequencing

Approximately 250 µg of soil or shell tissue per sample was used to isolate fungal DNA using the QIAGEN Power Soil DNA extraction kit (Cat No.12888-100) following the manufacturer’s protocols. At least 50 ng (10 μL) of DNA sample were sent to the Dalhousie University CGEB-IMR (https://imr.bio (accessed on 2 March 2022)) for V6–V8 16S rRNA gene (16S; forward: ACGCGHNRAACCTTACC; reverse: ACGGGCRGTGWGTRCAA) and fungal ITS2 region (ITS; forward: GTGAATCATCGAATCTTTGAA; reverse: TCCTCCGCTTATTGATATGC) library preparation and sequencing. Samples were multiplexed using a dual-indexing approach and sequenced using an Illumina MiSeq with paired-end 300 + 300 bp reads. All PCR procedures and Illumina sequencing details were as previously described [29,30]. All sequences generated in this study are available in the NCBI sequence read archive under the accession numbers PRJNA835461 and PRJNA835468.

### 2.3. Sequence Processing

The overlapping paired-end forward and reverse reads were stitched together using PEAR [31] and exported into QIIME2 [32]. The sequences were trimmed of their primers using QIIME2’s Cutadept plug-in [33,34]. Low-quality sequences were filtered from the dataset using QIIME2’s q-score-joined function. Using QIIME2’s Deblur plug-in, the sequences were organized into amplicon sequence variants (ASVs)–high resolution genomic groupings [33,35,36]. In order to account for the potential MiSeq bleed-through between runs (estimated by Illumina to be less than 0.1%), ASVs that accounted for less than 0.1% of the total sequences were removed. Taxonomic classifications were assigned to the ASV using QIIME2’s naïve-Bayes scikit-learn function, referencing SILVA databases [37,38]. Additionally, ASV assigned to mitochondria and chloroplasts were filtered out [33]. Sequencing of one 16S soil sample failed. After filtering unclassified and plant-derived ASVs, a total of 413,496 and 732,021 high-quality 16S and ITS reads were obtained from 44 and 45 samples, respectively. These reads were distributed across 4915 and 1003 16S and ITS ASVs, respectively. To assess microbial diversity, the sets were normalized to the depth of 3123 16S and 3758 ITS reads per sample, resulting in the identification of 4901 bacterial and 1001 fungal ASVs. During the normalization process, 2 ITS control and 4 16S soil samples were removed from analysis.

### 2.4. Bioinformatics and Statistical Analysis

QIIME2’s diversity function was used to calculate Shannon indices (alpha diversity) as well as UniFrac matrices (beta diversity) [39,40]. These UniFrac matrices were then subjected to an ADONIS test to determine what proportion of variance in the community structure could be attributed to treatment. Nonmetric multidimensional scaling (NMDS) of bacterial communities was performed on Bray–Curtis matrices using the Vegan R package [41]. Differential abundances in bacterial taxa were determined using ALDEx2 [42] with the Benjamini–Hochberg-corrected *p*-value of the Kruskal–Wallace test (*p* < 0.05). The graphics were produced using ggplot2 [43]. Core ASVs were identified using the QIIME2 core-features plugin. The co-occurrence analysis was performed using the CCREPE (Compositionality Corrected by REnormalization and PErmutation) R package [44] with 1000 bootstrap iterations and default settings. To obtain comparable datasets from each treatment, 10 replicate samples from the Control, Soil, and Shell dataset were randomly selected. The co-occurrence and co-exclusion patterns in the samples were scored. The results were filtered to remove nonstatistically significant relationships. We generated the network based on correlations with *p*-values < 0.05. The networks were analyzed with Cytoscape [45]. Indicator species (*p*-values < 0.001, computed using 5000 permutations) were determined by multi-level pattern analysis using R package “Indicspecies” [46].

## 3. Results

### 3.1. Taxonomic Compositing

Overall, 79 bacterial and 25 fungal classes were detected in the dataset (Figure S1). In the total microbiome, *Bacteroidia*, *Alphaproteobacteria*, *Gammaproteobacteria*, *Actinobacteria*, and *Clostridia* were the dominant bacterial classes (18%, 16%, 14%, 13%, and 8%, respectively) and *Mortierellomycetes*, *Sordariomycetes*, and *Leotiomycetes* were the dominant fungi (24%, 22%, and 14%, respectively). The Control, Soil, and Shell microbiomes showed very different taxonomic compositions. The Control microbiome was dominated by unclassified Ascomycota, followed by *Leotiomycetes*, *Dothideomycetes*, and *Sordariomycetes* (28%, 21%, 13%, and 12%, respectively), while the Soil and Shell microbiomes were dominated by *Sordariomycetes* (33% and 20%, respectively), *Mortierellomycetes* (16% and 50%, respectively), *Leotiomycetes* (14% and 7%, respectively), and *Eurotiomycetes* (12% and 7%, respectively) (Figure S1).

### 3.2. The Effect of Shell Proximity on the Diversity of Microbial Communities

**Figure 1:**
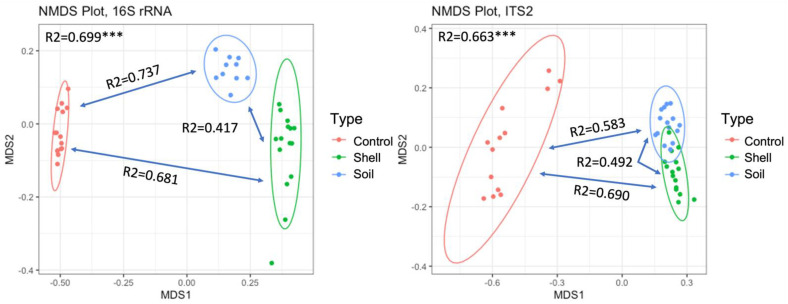
Nonmetric multidimensional scaling (NMDS) of bacterial and fungal communities at ASV level. The difference between communities based on Bray-Curtis distance Adonis tests was used to assess whether beta-diversity is related to sample groupings, 999 permutations, R2, *** *p* < 0.001.

**Figure 2:**
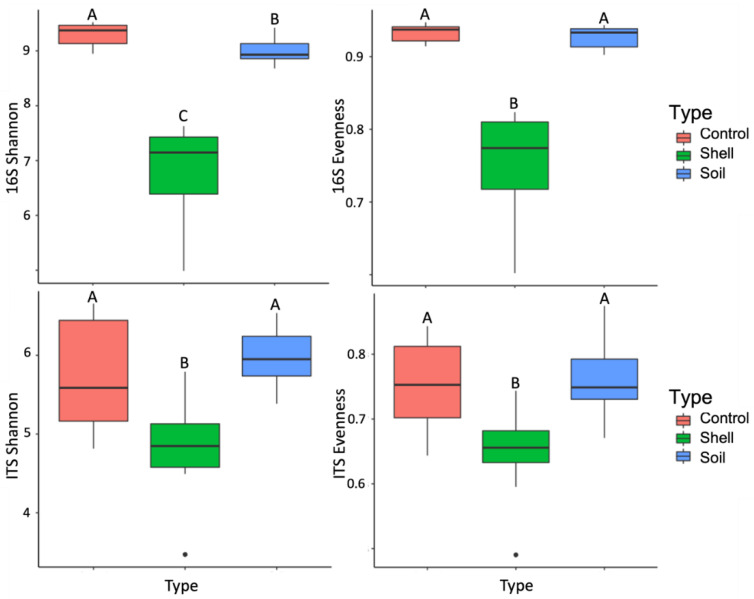
Estimated Shannon diversity and Evenness of bacterial and fungal communities. For each variable, data followed by different letters are significantly different according to Kruskal-Wallis pairwise test (*p* < 0.05).

We observed profound changes in microbial alpha- and beta-diversity with the increase in shell proximity. The niche, Control vs. Soil vs. Shell, explained 70% and 66% of bacterial and fungal communities’ variation, respectively (Table S1; Figure 1). Substantial variation was also detected between bacterial and fungal communities from Control vs. Soil groups (75% and 58%, respectively), Control vs. Shell samples (68% and 69%, respectively), and Soil vs. Shell samples (42% and 50%, respectively) (Table S1; Figure 1). The Shell microbiome exhibited a significant decrease in both bacterial and fungal Shannon diversity and Evenness, compared to the Control and the Soil microbiomes and bacterial Shannon diversity, which had a significant decrease in the Soil compared to the Control microbiome (Figure 2).

### 3.3. The Effect of Shell Proximity on the Composition of Microbial Communities

**Figure 3:**
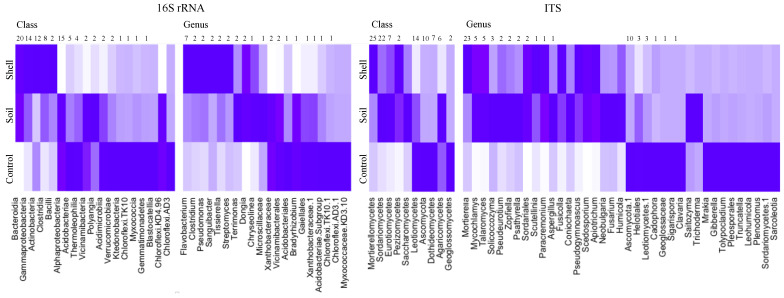
Bacterial and fungal taxa that were differentially represented between Shell, Soil, and Control microbiomes. Based on ALDEx2 Benjamini–Hochberg-corrected *p*-value of Kruskal–Wallace test.

The proximity of the shell had a dramatic effect on microbiome composition. A total of 55 bacterial and 17 fungal classes and 401 bacterial and 131 fungal genera were differentially represented between niches. Highly abundant fungal classes *Mortierellomycetes*, and *Pezizomycetes* and bacterial classes *Bacteroidia*, *Gammaproteobacteria*, *Actinobacteria*, *Clostridia*, and *Bacilli* were significantly enriched in the Shell microbiome. Considering highly abundant genera, bacteria *Flavobacterium*, *Clostridium*, *Pseudomonas*, *Sanguibacter*, *Tissierella*, and *Streptomyces* and fungi *Mortierella*, *Scutellinia*, and *Paracremonium* were enriched in the Shell microbiome (Figure 3).

Additionally, 430 bacterial and 173 fungal ASVs were differentially represented between the niches (Supplementary Tables S2 and S3), which represented 9% of 16S and 17% of ITS ASVs. These ASVs contained 55% and 75% of total 16S and ITS reads, respectively. The ASVs enriched in the Shell microbiome were represented by 47% and 63% of total 16S and ITS reads, respectively. Those highly abundant and overrepresented in the Shell microbiome ASVs were annotated as *Clostridium* (1 ASV, 6%), *Sanguibacter* (6%), *Streptomyces* (4%), *Flavobacterium* (3 ASVs: 3%, 2%, and 2%), *Pseudomonas* (2 ASVs: 2% and 2%), *Mortierella* (7%), *M. hyaline* (19%), *M. hypsicladia* (10%), *M. macrospora* (3%), *M. hypsicladia* (2 ASVs: 5% and 2%), *M. zonata* (3%), *Pseudeurotium hygrophilum* (2%), and *Talaromyces atroroseus* (2%). In total, these ASVs were represented by 27% and 51% to total 16S and ITS reads, respectively.

**Figure 4:**
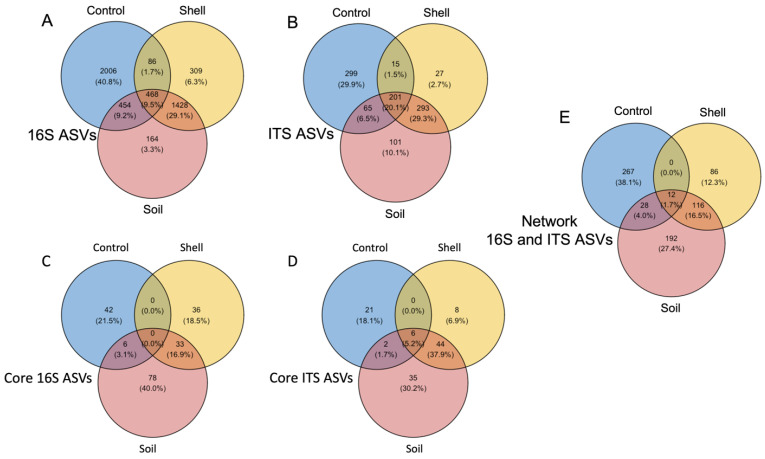
Venn diagrams showing the overlap between microbial communities from Shell, Soil, and Control microbiomes. (**A**) Bacterial and (**B**) fungal ASVs found in total microbiomes; (**C**) bacterial and (**D**) fungal ASVs found in core microbiomes; (**E**) bacterial and fungal ASVs found in network interaction.

The fungal community showed a greater overlap (>21%) in the ASVs between all three niches (Control, Soil, and Shell) compared with the bacterial community (<10%) (Figure 4A,B). The Control and the Shell microbiomes had minor overlap (<2%). The Soil microbiome shared more ASVs with the Shell compared to the Control microbiome—<10% of bacteria and <7% of fungal ASVs were shared between the Soil and Control, while >29% of both fungal and bacterial ASVs were shared between the Soil and Shell microbiomes. For both bacterial and fungal communities, the greatest proportion of specialists was found in the Control microbiome (41% and 39%, respectively). The shell microbiome specialists were represented by 6% and 3% of bacterial and fungal ASVs, respectively, and Soil microbiome specialists were represented by 3% and 10% of bacterial and fungal ASVs, respectively (Figure 4A,B).

### 3.4. Core Microbiomes

Next, we looked at core microbiomes in the Control, Soil, and Shell microbiomes, which was defined as ASVs found in all but one sample from each niche, which represented at least 92.5% of all samples in each niche (Supplementary Tables S4–S9). In general, both bacterial and fungal core microbiomes from the Control microbiome contained less ASVs compared to those from the Shell and Soil microbiomes. The bacterial Shell, Soil, and Control core microbiomes were composed of 69, 117, and 48 ASVs, respectively, and fungal core microbiomes contained 58, 87, and 29 ASVs, respectively (Figure 4C,D). The Shell and Soil core microbiomes shared the largest proportion of ASVs (17% of bacterial and 38% of fungal ASVs, respectively) compared to those shared between Soil and Control core microbiomes (3% of bacterial and 2% of fungal ASVs, respectively) (Figure 4C,D). In addition, 5% of fungal ASVs were shared between all three niches and no bacterial ASVs were common for these niches. Additionally, no ASVs were common between bacterial and fungal Control and Shell core microbiomes.

**Figure 5:**
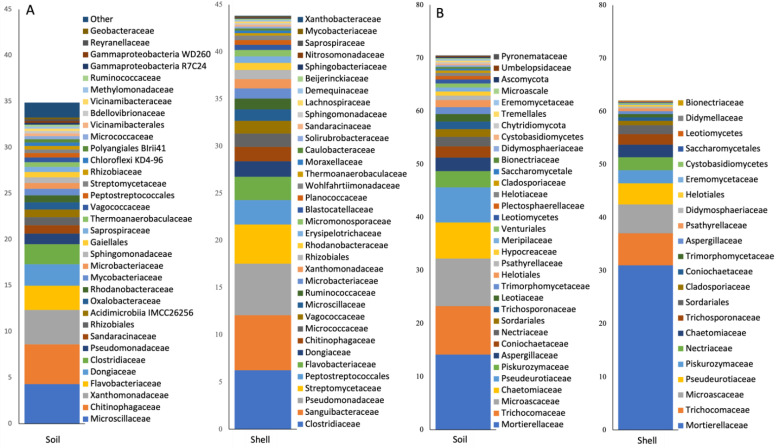
Microbial families identified in the core microbiomes. (**A**) Bacterial 16S rRNA; (**B**) fungal ITS.

When the relative abundances of core ASVs were combined together at the family taxonomic level, we found that the Shell core was dominated by a few bacterial and fungal families (Figure 5) such as bacteria *Clostridiaceae*, *Sanguibacteraceae*, *Pseudomonadaceae*, *Streptomycetaceae*, *Peptostreptococcales*, and *Flavobacteriaceae*, and fungi *Mortierellaceae*, *Trichocomaceae*, *Microascaceae*, *Pseudeurotiaceae*, *Piskurozymaceae*, and *Nectriaceae* (Figure 5). This families contained several taxa overrepresented in the overall Shell microbiome (Figure 3). Together, these families comprised 61% and 82% of the total 16S and ITS Shell core reads, respectively. These families were also found in Soil core microbiomes and were represented by 22% and 63% of total 16S and ITS Soil core reads, respectively. On the other hand, only two of these bacterial (*Clostridiaceae* and *Streptomycetaceae*) and fungal (*Mortierellaceae* and *Nectriaceae*) taxa were found in the Control core microbiome, and they were represented by 2% and 10% of total 16S and ITS Control core reads, respectively.

### 3.5. Microbiome Cooperation

We generated a co-occurrence network by correlating relative abundances between bacterial and fungal ASVs from the Control, Soil, and Shell microbiomes. The list of ASVs found in each network and their characteristics can be found in Supplementary Tables S10–S12. The Shell and Soil network exhibited less cooperation compared to the Control network. The Shell co-occurrence network contained 214 ASVs, 884 interactions, and an average of 8.262 neighbor ASVs, and the Soil co-occurrence network contained 348 ASVs, 1606 interactions, and an average of 9.230 neighbors, while the Control network interaction contained 307 ASVs, 2689 interactions, and an average of 17.518 neighbors. We found a significant overlap between the Shell and Soil network—more than half of the ASVs in the Shell network were also part of the Soil network (116 ASVs) and 12 ASVs were common to all three networks. Several most connected in the Shell network ASVs also found in the Soil network included *Dongia* (30 and 12 interaction), *Rhizobiales* (29 and 7 interaction), 2 *Tissierella* ASVs (29/22 and 7/4 interaction), *Mortierella hypsicladia* (28 and 9 interaction); *Scutellinia vitreola* (27 and 10 interaction), and *Mortierella* (24 and 7 interaction) in the Shell and Soil network, respectively. On the other hand, the Control network did not have much commonality with the Shell and Soil networks: it had no unique overlap with the Shell and had only 28 ASVs of unique overlap with the Soil network (Figure 4E).

### 3.6. 16S and ITS2 ASVs with Strong Association with Shell Environment

To identify the most influential taxonomic groups associated with the shell environments, we selected the Shell core ASVs with increased relative abundances in the Shell compared to the Control microbiome and filtered out the ASVs that were not part of the Shell network interaction (Supplementary Tables S13 and S14). These selected ASVs did not belong to the Control core microbiome and network interaction. We also analyzed the strength and statistical significance of the relationship between the ASVs occurrence/abundance and their association with the specific niche and verified that all selected ASVs were indicator species of the Shell/Soil microbiome. As a result, 60 bacterial and 44 fungal ASVs comprising 38% and 56% of Shell microbiome 16S and ITS reads, respectively, were selected. These ASVs were collapsed to 45 bacterial and 27 fungal genera or taxa with lower annotation levels above the genus (Table 1). The most abundant bacterial taxa included *Actinobacteria*, *Gamma-proteobacteria Pseudomonas*, *Bacteroidia Flavobacterium* and *Chryseolinea*, *Clostridia Tissierella* and *Ruminococcaceae*, *Alpha-proteobacteria Dongia*, and *Bacilli Vagococcus*. The most abundant fungal taxa included *Mortierellomycetes Mortierella*, *Eurotiomycetes Talaromyces*, *Sordariomycetes Mycochlamys*, *Fusicolla, Sordariales* and *Zopfiella*, *Leotiomycetes Pseudeurotium* and *Pseudogymnoascus*, and *Tremellomycetes Apiotrichum*. Together, they were represented by 25% and 50% of 16S and ITS reads in the Shell microbiome, respectively (Table 1). Fungi *Mortierella* were represented by 31% of Shell microbiome ITS reads and contained four species, *M. hyaline* (19%), *M. zonata* (3%), *M. hypsicladia* (0.9%), and *M. humilis* (0.6%) and annotated *Mortierella* (7%) (Supplementary Tables S13 and S14).

## 4. Discussion

A deep understanding of the process underlying the biological degradation of crustacean’s shell waste is an important step to maximize the utilization of its valuable components into high-value-added products and to facilitate environmentally friendly waste disposal. In this study, we explored microbial communities tightly associated with green crab shells composted in the soil for over a one-year period to identify the microbial complex of potential chitin degraders. To gain an overall understanding of the microbial community involved in shell composting in soil, we did not separate between the microbiomes located in microporosities, inside shell matter, in the shell surface, and in the biofilm state. We detected profound changes in microbial structure and composition with the increase in shell proximity. Both bacterial and fungal Shannon diversity and Evenness were significantly decreased in the Shell microbiome compared to adjacent (Soil) and distantly located (Control) soils. We detected a small but statistically significant decrease in bacterial Shannon diversity in the Soil compared to the Control. The decrease in the Shell microbiome alpha-diversity might reflect its strong specialization and enrichment with a relatively small set of taxa capable of obtaining nutrients from chitin matter or feed on chitin degradation products. Our results are consistent with a previous study that reported a decrease in diversity indices in the soils treated with chitin-rich amendments after 3 weeks post-incubation [8]. On the other hand, another ecological study reported an increase in the bacterial diversity after chitin enrichment after 35 days post-inoculation [21], indicating that our understanding of the effects of chitin on soil biology is far from complete.

The changes in microbiome composition with the increase in shell proximity were reflected in the differential representation of microbial taxa between niches. Our analysis identified a large number of ASVs differentially represented between the Control and Shell microbiomes. Interestingly, 47% and 63% of total 16S and ITS reads, respectively, represented ASVs with increased relative abundance in the Shell compared to Control microbiome, indicating that the adaptation of the microbiome to shell environment involved major microbial taxa. For example, highly relatively abundant bacterial genera *Flavobacterium*, *Clostridium*, *Pseudomonas*, *Sanguibacter*, *Tissierella*, *Streptomyces*, *Mortierella*, *Scutellinia*, and *Paracremonium* were enriched in the Shell microbiome. Previously, it was shown that *Flavobacterium*, *Pseudomonas*, *Sanguibacter*, *Streptomyces*, and fungi *Mortierella* and *Aspergillus* were representative genera of chitinolytic microbes [13,20,21,22,47,48]. On the other hand, to the best of our knowledge, there are no reports indicating the effect of chitin amendments on the abundances of bacteria *Tissierella* and fungi *Scutellinia* and *Paracremonium* in soil. It is possible that these microorganisms might not possess any chitinolytic activities but could take an advantage of chitin degradation products available in the shell environment. Another possibility is that the previous studies were looking at microorganisms associated with chitin-amended soil, while our study was focused on the shell matter with very different physical and chemical properties, which might have provided additional selective pressure for enrichment of chitinolytic microorganisms. The enrichment in *Clostridium* spp. might be linked to the activation of fermentation processes in the Shell microbiome. We also observed a significant overlap between the Shell and Soil microbiomes, and about 75% of 16S and ITS Soil ASVs were shared with the Shell microbiome. On the other hand, only 18% of 16S and 37% of ITS ASVs were shared between the Control and Shell microbiomes, indicating a significant influence of shell matter on the composition of the adjacent soil microbiome.

The above conclusion was also supported by the fact that 48% of 16S and 76% of ITS Shell core ASVs were shared with the Soil core, while only 1% of ITS Shell core ASVs were shared with the Control core microbiome and none of the 16S Shell core ASVs were found in the Control core. Additionally, the six most abundant bacterial and fungal classes found in the Shell core microbiome were represented by 61% and 82% of total 16S and ITS Shell core reads, respectively, reiterating our previous conclusion of the involvement of the major taxa in microbial adaptation to the shell environment.

Microbial cooperation was also affected by shell proximity, which was reflected in the decrease in microbial cooperation in the Shell and Soil compared to the Control network. This suggested a stronger specialization within the shell-associated microbiome involving a limited number of microbial taxa capable of directly feeding on the shell matter or to benefit from chitin degradation products available in the shell environment. We also detected a strong overlap between the Soil and the Shell networks. Around 60% of microbial ASVs from the Shell network were part of the Soil network, which might be a result of a significant enrichment of adjacent soil with byproducts of shell degradation stimulating a similar type of cooperation between microorganisms. However, we also should consider a possibility that small shell particles could be present in the Soil samples and therefore influence the diversity and structure of the Soil microbiome.

In our study, we applied the analyses of taxonomic composition, core microbiome, and network cooperation to identify ASVs potentially involved in shell degradation in soil. These analyses produced 3 groups of ASVs, which could be involved in this process based on a single defined criterion. However, some of these ASVs were identified as important based on more than one criterion, while others were only found in one of the groups. We speculated that the ASVs found in the overlap between all three groups could be considered as the most active participants in the shell degradation consortium. This approach allowed us to select 60 bacterial and 44 fungal ASVs comprising 45 bacterial and 27 fungal genera. These ASVs represented a tiny proportion of ASVs identified in the Shell microbiome (3% and 8% of 16S and ITS ASVs, respectively) but comprised a large proportion of 16S and ITS Shell microbiome reads (38% and 56%, respectively), again emphasizing the involvement of major ASVs in shell degradation.

Major bacterial classes identified by this approach included *Actinobacteria*, *Gamma-proteobacteria*, *Bacteroidia*, and *Clostridia*, represented by 13%, 8%, 5%, and 5% of Shell microbiome reads. Well-known chitinolytic bacteria *Sanguibacter* (6%) and *Streptomyces* (4%) [7,20,49] were the most abundant genera from class *Actinobacteria. Gamma-proteobacteria Pseudomonas caeni* and *Bacteroidia Flavobacterium* were represented by 5% and 2% of Shell microbiome reads, respectively. It was reported that some *Pseudomonas and Flavobacterium* spp. possess chitinolytic activities [22,47,50,51,52,53,54] and have been used for chitin extraction [55,56]. Highly abundant *Clostridia Tissierella* (2%) and *Lactobacillales Vagococcus* are known fermenters [57,58,59,60]. *Tissierella* was also identified as one of the most connected taxa in network interaction, which underlined the importance of this bacteria in shell degradation.

The most active fungi in the shell degradation consortium included *Mortierella* (30%), *Talaromyces* (5%), *Mycochlamys* (4%), *Fusicolla* (2%), *Sordariales* (2%), and *Pseudeurotium* (2%). It has been reported that some *Mortierella* spp. have a chitinolytic activity [47] and their abundance is positively correlated with soil chitin [9]. In our study, we also detected a substantial increase in relative abundance of *Mortierella* in the Shell compared to the Control microbiome. Two most dominant *Mortierella* spp. found in the consortiums, *M. hyaline* (19%) and *M. zonata* (3%), were reported to possess a strong plant growth-promoting capability [61,62]. Additionally, *Mortierella* spp. and *Flavobacteriaceae* spp. have been correlated with disease suppression [63], emphasizing the potential of shell-fish waste-based amendments in the manipulation of the soil mycobiome to promote disease suppression. There are several reports on the isolation of chitinases from *Talaromyces* spp., a highly abundant genus found in the shell degradation consortium [64,65,66]. However, as far as we know, the direct involvement of genera *Fusicolla*, *Mycochlamys,* and *Pseudeurotium* in chitin degradation has not been documented. Nevertheless, there was a report indicating an increase in relative abundances of *Mycochlamys* and *Pseudeurotium* in chitin-treated soil [13], and *Fusicolla* has been identified as part of the sea-food fermentation microbiome [67]. Given the active involvement of these fungi in the shell degradation consortium and their high abundances in the Shell and Soil microbiomes, the additional study of the role on these taxa in chitin degradation should be examined in greater detail.

## 5. Conclusions

In the present study, we demonstrated profound changes in microbial structure and composition with the increase in shell proximity, which was reflected in the enrichment of the Shell microbiome with the subset of taxa with potential to obtain nutrients from chitin matter or feed on chitin degradation products. Our analysis identified a large number of microbial taxa overrepresented in the Shell compared to the Control microbiome. We applied a combination of several bioinformatic tools to narrow down this list of microorganisms to focus on the most active participants in the shell degradation consortium. This approach allowed us to identify a group of microbes that might play a central role in the degradation of shell matter in soil and might form tightly cooperated consortiums to facilitate nutrient acquisition from shell matter. In addition to a number of microorganisms previously reported to have chitin degradation activities, several genera without a known role in this process were identified in the consortiums. These taxa might play an active role in chitin degradation or take advantage of degradation products available in the shell environment and therefore facilitate chitin degradation. These newly identified microorganisms should be further studied for their potential application in environmentally friendly chitin degradation.

## Figures and Tables

**Table 1:** 16S rRNA and ITS ASVs with potential to be directly involved in shell degradation.

Genera/Lowest Annotation	Total *	Control	Soil	Shell	Taxonomy
**16S ASVs**					
Sanguibacter	3.095	0.000	0.272	5.820	Actinobacteriota Actinobacteria Micrococcales Sanguibacteraceae
Pseudomonas, 4 **	3.140	0.006	1.738	5.456	Proteobacteria Gammaproteobacteria Pseudomonadales Pseudomonadaceae
Streptomyces, 2	2.247	0.000	0.428	4.156	Actinobacteriota Actinobacteria Streptomycetales Streptomycetaceae
Flavobacterium	1.380	0.000	0.557	2.462	Bacteroidota Bacteroidia Flavobacteriales Flavobacteriaceae
Tissierella, 4	1.165	0.000	0.641	2.026	Firmicutes Clostridia Peptostreptococcales-Tissierellales
Dongia, 2	1.182	0.000	1.980	1.651	Proteobacteria Alphaproteobacteria Dongiales Dongiaceae
Vagococcus	0.792	0.000	0.499	1.358	Firmicutes Bacilli Lactobacillales Vagococcaceae
Ruminococcaceae	0.628	0.000	0.212	1.132	Firmicutes Clostridia Oscillospirales
Chryseolinea	0.935	0.000	2.219	1.108	Bacteroidota Bacteroidia Cytophagales Microscillaceae
Micrococcaceae	0.516	0.000	0.331	0.884	Actinobacteriota Actinobacteria Micrococcales
Clostridium, 2	0.475	0.000	0.655	0.706	Firmicutes Clostridia Clostridiales Clostridiaceae
Erysipelothrix	0.410	0.000	0.275	0.697	Firmicutes Bacilli Erysipelotrichales Erysipelotrichaceae
Stenotrophomonas	0.346	0.000	0.068	0.640	Proteobacteria Gammaproteobacteria Xanthomonadales Xanthomonadaceae
Microbacteriaceae, 2	0.357	0.002	0.144	0.635	Actinobacteriota Actinobacteria Micrococcales
Terrimonas	0.406	0.000	0.672	0.570	Bacteroidota Bacteroidia Chitinophagales Chitinophagaceae
Chitinophagaceae	0.334	0.000	0.225	0.569	Bacteroidota Bacteroidia Chitinophagales
Longispora	0.300	0.000	0.156	0.525	Actinobacteriota Actinobacteria Micromonosporales Micromonosporaceae
Rhizobiales, 2	0.336	0.000	0.464	0.499	Proteobacteria Alphaproteobacteria
Ignatzschineria, 2	0.279	0.000	0.144	0.488	Proteobacteria Gammaproteobacteria Cardiobacteriales Wohlfahrtiimonadaceae
Leifsonia, 2	0.291	0.000	0.307	0.461	Actinobacteriota Actinobacteria Micrococcales Microbacteriaceae
Dokdonella	0.2	0.000	0.089	0.429	Proteobacteria Gammaproteobacteria Xanthomonadales Rhodanobacteraceae
Peptostreptococcus	0.245	0.000	0.141	0.424	Firmicutes Clostridia Peptostreptococcales-Tissierellales Peptostreptococcaceae
Rhodanobacteraceae	0.174	0.000	0.056	0.316	Proteobacteria Gammaproteobacteria Xanthomonadales
Pseudarthrobacter	0.180	0.000	0.138	0.301	Actinobacteriota Actinobacteria Micrococcales Micrococcaceae
Lysinibacillus	0.151	0.000	0.003	0.286	Firmicutes Bacilli Bacillales Planococcaceae
Blastocatellaceae	0.159	0.000	0.103	0.273	Acidobacteriota Blastocatellia Blastocatellales
Lysobacter	0.156	0.000	0.082	0.272	Proteobacteria Gammaproteobacteria Xanthomonadales Xanthomonadaceae
Bryobacter	0.164	0.000	0.154	0.267	Acidobacteriota Acidobacteriae Bryobacterales Bryobacteraceae
Subgroup 10	0.195	0.000	0.372	0.259	Acidobacteriota Thermoanaerobaculia Thermoanaerobaculales Thermoanaerobaculaceae
Psychrobacter	0.148	0.000	0.077	0.258	Proteobacteria Gammaproteobacteria Pseudomonadales Moraxellaceae
Shinella	0.140	0.000	0.050	0.251	Proteobacteria Alphaproteobacteria Rhizobiales Rhizobiaceae
Flavihumibacter	0.230	0.000	0.622	0.249	Bacteroidota Bacteroidia Chitinophagales Chitinophagaceae
Phenylobacterium, 2	0.132	0.000	0.050	0.237	Proteobacteria Alphaproteobacteria Caulobacterales Caulobacteraceae
Paeniglutamicibacter	0.129	0.000	0.054	0.230	Actinobacteriota Actinobacteria Micrococcales Micrococcaceae
Psychrobacillus	0.113	0.000	0.012	0.212	Firmicutes Bacilli Bacillales Planococcaceae
Solirubrobacteraceae	0.156	0.000	0.328	0.198	Actinobacteriota Thermoleophilia Solirubrobacterales
Sandaracinus	0.106	0.000	0.035	0.192	Myxococcota Polyangia Polyangiales Sandaracinaceae
Methylomonadaceae	0.130	0.000	0.219	0.182	Proteobacteria Gammaproteobacteria Methylococcales
Lachnospiraceae	0.102	0.000	0.068	0.173	Firmicutes Clostridia Lachnospirales
Allocatelliglobosispora	0.079	0.000	0.003	0.149	Actinobacteriota Actinobacteria Micromonosporales Micromonosporaceae
Romboutsia	0.099	0.000	0.141	0.147	Firmicutes Clostridia Peptostreptococcales-Tissierellales Peptostreptococcaceae
Bosea	0.076	0.000	0.048	0.130	Proteobacteria Alphaproteobacteria Rhizobiales Beijerinckiaceae
Flavitalea	0.072	0.000	0.041	0.125	Bacteroidota Bacteroidia Chitinophagales Chitinophagaceae
Sphingomonadaceae	0.087	0.000	0.145	0.122	Proteobacteria Alphaproteobacteria Sphingomonadales Sphingomonadaceae
Microscillaceae	0.107	0.000	0.316	0.108	Bacteroidota Bacteroidia Cytophagales
Pedobacter	0.057	0.000	0.061	0.090	Bacteroidota Bacteroidia Sphingobacteriales Sphingobacteriaceae
**ITS2 ASVs**					
Mortierella, 5	16.213	0.804	9.626	30.505	Mortierellomycota Mortierellomycetes Mortierellales Mortierellaceae
Talaromyces, 8	4.836	0.006	6.995	5.054	Ascomycota Eurotiomycetes Eurotiales Trichocomaceae
Mycochlamys, 2	4.784	0.003	7.923	3.990	Ascomycota Sordariomycetes Microascales Microascaceae
Pseudeurotium	2.998	0.028	5.280	2.169	Ascomycota Leotiomycetes Thelebolales Pseudeurotiaceae
Fusicolla, 2	1.204	0.020	1.082	1.914	Ascomycota Sordariomycetes Hypocreales Nectriaceae
Sordariales	1.157	0.006	1.195	1.691	Ascomycota Sordariomycetes
Pseudogymnoascus	1.017	0.028	0.944	1.583	Ascomycota Leotiomycetes Thelebolales Pseudeurotiaceae
Zopfiella	2.651	0.000	5.046	1.549	Ascomycota Sordariomycetes Sordariales Chaetomiaceae
Apiotrichum	0.724	0.006	0.708	1.096	Basidiomycota Tremellomycetes Trichosporonales Trichosporonaceae
Scedosporium	0.456	0.000	0.237	0.905	Ascomycota Sordariomycetes Microascales Microascaceae
Trichosporonaceae	0.365	0.003	0.156	0.756	Basidiomycota Tremellomycetes Trichosporonales
Coniochaeta, 2	1.118	0.026	2.129	0.641	Ascomycota Sordariomycetes Coniochaetales Coniochaetaceae
Aspergillus, 3	1.250	0.001	2.569	0.538	Ascomycota Eurotiomycetes Eurotiales Aspergillaceae
Coprinopsis	0.542	0.000	0.827	0.525	Basidiomycota Agaricomycetes Agaricales Psathyrellaceae
Humicola	0.604	0.000	1.049	0.456	Ascomycota Sordariomycetes Sordariales Chaetomiaceae
Rasamsonia	0.443	0.000	0.695	0.408	Ascomycota Eurotiomycetes Eurotiales Trichocomaceae
Paracremonium	0.407	0.000	0.616	0.399	Ascomycota Sordariomycetes Hypocreales Nectriaceae
Parascedosporium	0.308	0.000	0.467	0.300	Ascomycota Sordariomycetes Microascales Microascaceae
Cephalotrichum, 2	0.212	0.000	0.278	0.252	Ascomycota Sordariomycetes Microascales Microascaceae
Thermomyces	0.421	0.000	0.810	0.237	Ascomycota Eurotiomycetes Eurotiales Trichocomaceae
Pseudeurotiaceae	0.254	0.000	0.407	0.226	Ascomycota Leotiomycetes Thelebolales
Anguillospora	0.329	0.021	0.584	0.224	Ascomycota Leotiomycetes Helotiales Helotiaceae
Arthrographis	0.181	0.001	0.249	0.201	Ascomycota Dothideomycetes Eremomycetaceae
Cystobasidiomycetes	0.191	0.000	0.295	0.182	Basidiomycota
Candida	0.204	0.008	0.343	0.162	Ascomycota Saccharomycetes Saccharomycetales
Leotiomycetes	0.275	0.000	0.529	0.155	Ascomycota
Sugiyamaella	0.344	0.007	0.707	0.144	Ascomycota Saccharomycetes Saccharomycetales Trichomonascaceae

* Relative abundance in percent (%) of 16S and or ITS2 reads in the specific niche and ** Number of ASVs with same taxonomic annotation.

## Data Availability

Data can be found within article and in supplementary files.

## Supplementary Materials

The following supporting information can be downloaded at: https://www.mdpi.com/article/10.3390/microorganisms10051033/s1. Figure S1: Microbial classes identified in the study. A, bacterial 16S rRNA; B, fungal ITS; Figure S2: Microbial families identified in the core microbiomes. A, bacterial 16S rRNA; B, fungal ITS; Table S1: Variation in sample groupings as explained by weighted UniFrac dissimilarity distances. Adonis tests were used to assess whether beta-diversity is related to sample groupings, 999 permutations, R2, *** *p* < 0.001; Table S2: Bacterial ASVs that showed significant differences in relative abundance between Shell, Soil, and Control microbiomes based on ALDEx2. kw.ep—Expected *p*-value of Kruskal–Wallace test; kw.eBH—Expected Benjamini–Hochberg-corrected *p*-value of Kruskal–Wallace test; glm.ep—Expected *p*-value of glm test; glm.eBH—Expected Benjamini–Hochberg-corrected *p*-value of glm test. In bold are the ASVs found significantly overrepresented based on kw.eBH; Table S3: Fungal ASVs that showed significant differences in relative abundance between Shell, Soil, and Control microbiomes based on ALDEx2. kw.ep—Expected *p*-value of Kruskal–Wallace test; kw.eBH—Expected Benjamini–Hochberg-corrected *p*-value of Kruskal–Wallace test; glm.ep—Expected *p*-value of glm test; glm.eBH—Expected Benjamini–Hochberg-corrected *p*-value of glm test. In bold are the ASVs found significantly overrepresented based on kw.eBH; Table S4: Bacterial ASVs identified in core Shell microbiome; Table S5. Fungal ASVs identified in core Shell microbiome; Table S6: Bacterial ASVs identified in core Soil microbiome; Table S7: Fungal ASVs identified in core Soil microbiome; Table S8: Bacterial ASVs identified in core Control microbiome; Table S9: Fungal ASVs identified in core Control microbiome; Table S10: Node statistics for network representing cooperation in Shell microbiome; Table S11: Node statistics for network representing cooperation in Soil microbiome; Table S12: Node statistics for network representing cooperation in Control microbiome; Table S13: Bacterial ASVs with potential to be directly involved in shell degradation; Table S14: Fungal ASVs with potential to be directly involved in shell degradation.
