# Supplementary material for: Microbial Consortium Associated with Crustacean Shells Composting
Source: Microorganisms. 2022 May 16;10(5):1033. doi: 10.3390/microorganisms10051033 (PMC9145653; doi:10.3390/microorganisms10051033)
Supplement: Supplementary file 1 [file microorganisms-10-01033-s001.zip › Supplementary_Material_Final.pdf]

Supplementary Figures

Figure S1: Microbial classes identified in the study. A, bacterial 16S rRNA; B, fungal ITS.

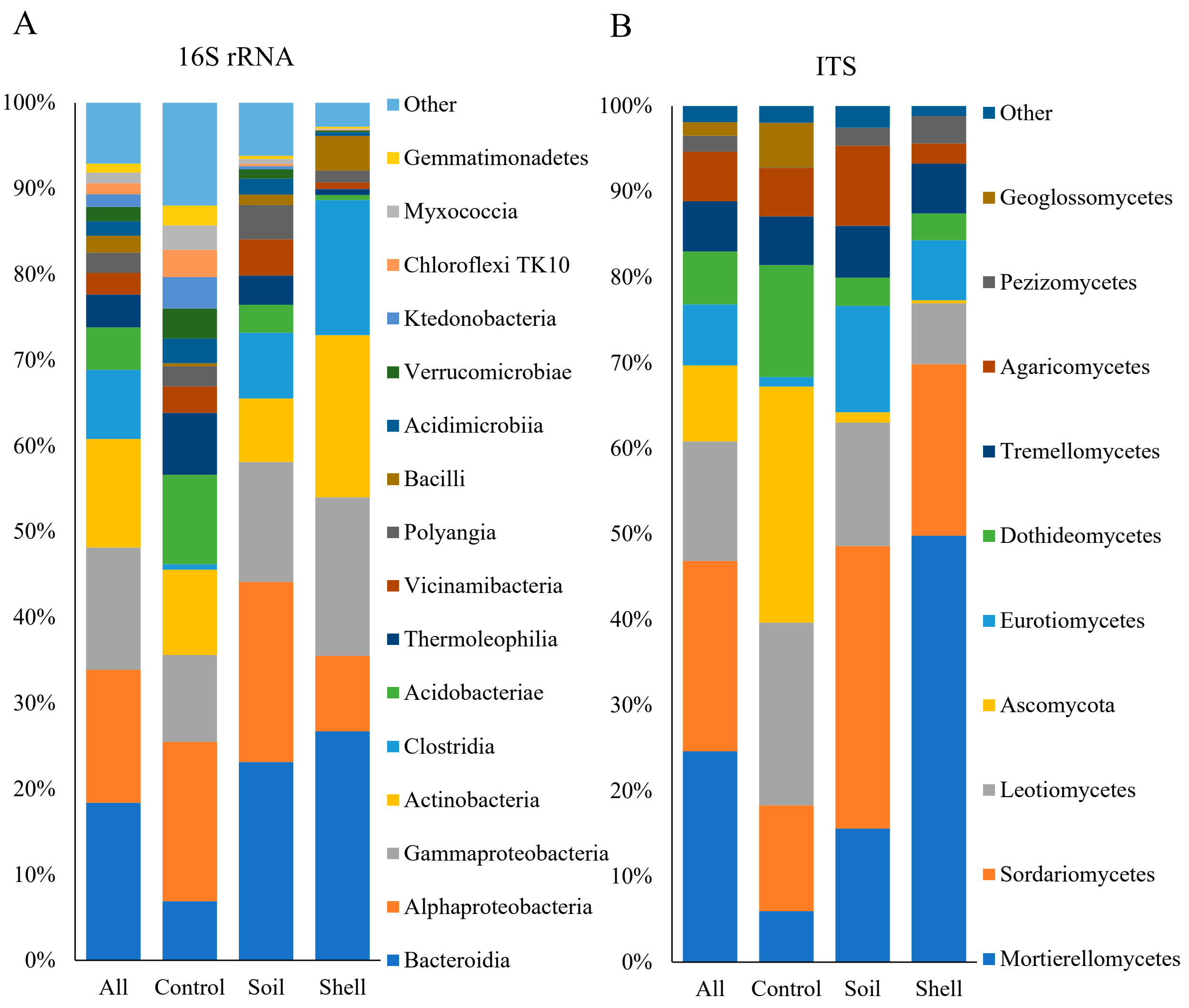

Figure S2: Microbial families identified in the core microbiomes. A, bacterial 16S rRNA; B, fungal ITS.

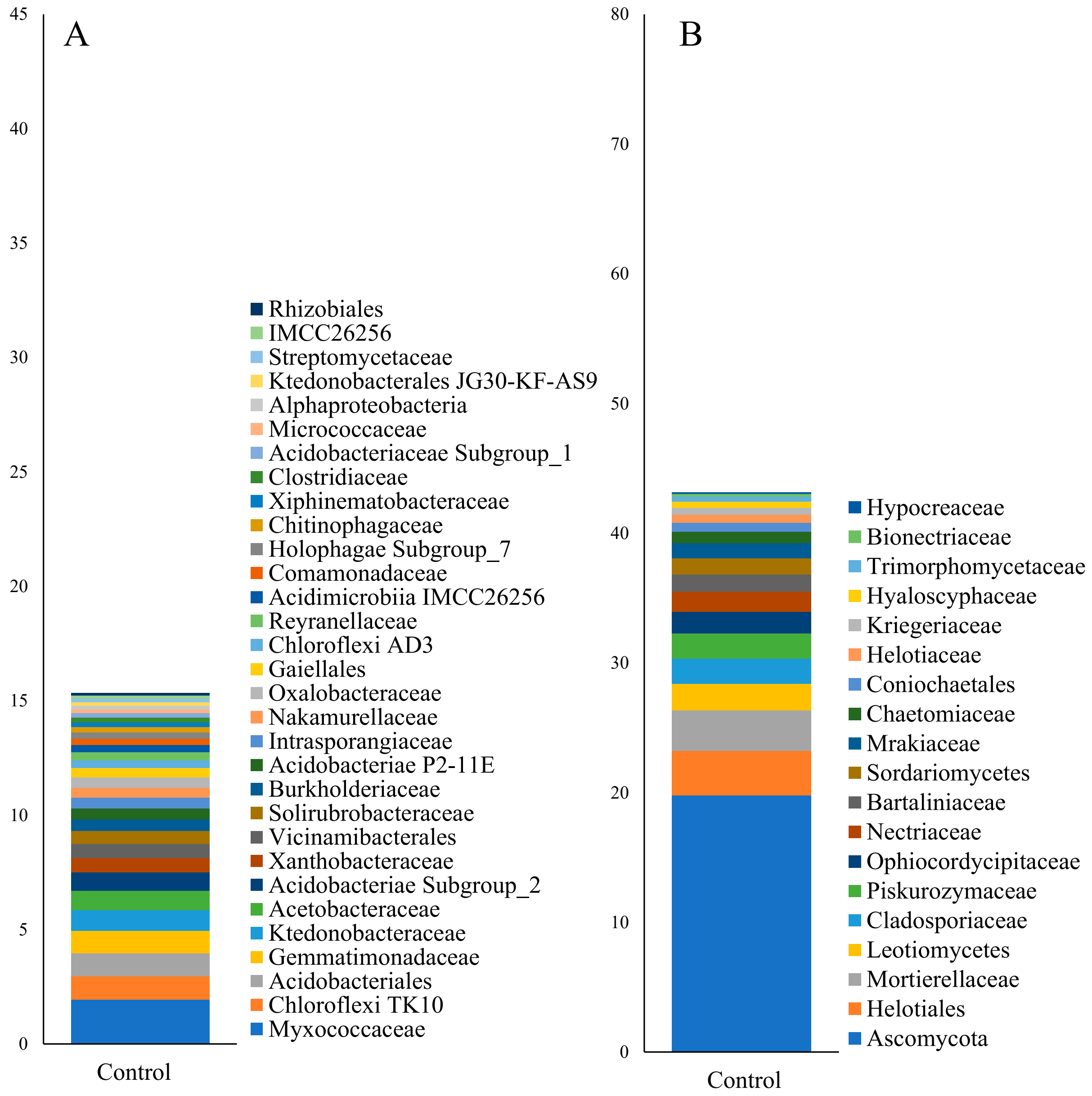

Supplementary Tables

Table S1: Variation in sample groupings as explained by weighted UniFrac dissimilarity distances.

	**16S rRNA**	**ITS2**
**Control x Soil x Shell**	0.699***	0.663***
**Control x Soil**	0.737***	0.583***
**Control x Shell**	0.681***	0.690***
**Soil x Shell**	0.417***	0.492***

*Adonis tests were used to assess whether beta-diversity is related to sample groupings, 999 permutations, R2, ***P < 0.001.*
